# 2-kupl: mapping-free variant detection from DNA-seq data of matched samples

**DOI:** 10.1186/s12859-021-04185-6

**Published:** 2021-06-05

**Authors:** Yunfeng Wang, Haoliang Xue, Christine Pourcel, Yang Du, Daniel Gautheret

**Affiliations:** 1 Institute of Integrative Cell Biology (I2BC), Université Paris-Saclay, CNRS, CEA, 1 avenue de la Terrasse, 91190 Gif-sur-Yvette, France; 2 IHU PRISM, Gustave Roussy, 114 rue Edouard Vaillant, 94800 Villejuif, France; 3 Annoroad Gene Technology Co., Ltd, Beijing, 100176 China

**Keywords:** DNAseq, WGS, WES, k-mers, Contigs, Recurrent variants, PRAD, Mapping-free

## Abstract

**Background:**

The detection of genome variants, including point mutations, indels and structural variants, is a fundamental and challenging computational problem. We address here the problem of variant detection between two deep-sequencing (DNA-seq) samples, such as two human samples from an individual patient, or two samples from distinct bacterial strains. The preferred strategy in such a case is to align each sample to a common reference genome, collect all variants and compare these variants between samples. Such mapping-based protocols have several limitations. DNA sequences with large indels, aggregated mutations and structural variants are hard to map to the reference. Furthermore, DNA sequences cannot be mapped reliably to genomic low complexity regions and repeats.

**Results:**

We introduce 2-kupl, a k-mer based, mapping-free protocol to detect variants between two DNA-seq samples. On simulated and actual data, 2-kupl achieves higher accuracy than other mapping-free protocols. Applying 2-kupl to prostate cancer whole exome sequencing data, we identify a number of candidate variants in hard-to-map regions and propose potential novel recurrent variants in this disease.

**Conclusions:**

We developed a mapping-free protocol for variant calling between matched DNA-seq samples. Our protocol is suitable for variant detection in unmappable genome regions or in the absence of a reference genome.

**Supplementary Information:**

The online version contains supplementary material available at 10.1186/s12859-021-04185-6.

## Background

Searching for genomic variants is a fundamental aspect of medical research, whether in the study of Mendelian diseases or of somatic, cancer-related alterations [1]. While certain variants result in gene dysfunction and disease [2], others are largely asymptomatic but give rise to neoantigens relevant to immune escape and therapeutic efficacy or treatment [3]. Genome variants are also of interest in microbiology to analyze the differences between microbial strains [4] and reveal mechanisms underlying phenotypes. In this study, we address the problem of finding genomic differences between a matching pair of high throughput DNA sequencing (DNA-seq) datasets from the same individual (human somatic variation) or from two bacterial strains.

Genomic variants include mutations, indels and structural variants (SV). Mutations and indels can alter genes by disrupting the genetic code, while SVs, by pulling distant regions together or splitting one region into segments, can create chimeric genes or have a broader impact on whole chromosomal regions [5]. Variants are typically detected by whole-genome (WGS) or whole-exome (WES) sequencing through comparison with reference sequences. Aligners such as BWA [6] are first applied to map reads to the reference sequences. The variant calling step then detects differences between mapped reads and the reference. Popular variant callers include MuTect2 [7], VarScan [8], somaticsniper [9] and MuSE [10]. Based on variants observed between two sequence samples and a common reference genome, these programs can then infer differences between the two samples (e.g., in MuTect2’s somatic mode).

Reference-based variant calling has well-known limitations. Aligners may encounter difficulties while handling reads with low mapping qualities [11], originating from repeat regions, low complexity regions or complex variants. These reads of low mapping quality are usually discarded. Furthermore, some species have no reliable reference, which is common in microbes [12].

Alternative approaches to variant calling involve mapping-free protocols [13]. These methods do not rely on a reference genome and can directly predict variants from the raw fastq file. A typical strategy is to use a de Bruijn graph (DBG) [14]. A DBG is constructed using k-mers (subsequences of fixed size k) decomposed from the sequence reads. The occurrence of k-mers harboring a mutant allele and a wild type allele generates a bubble structure in the DBG. Variant callers developed based on DBGs include DiscoSNP++ [15] and Lancet [16]. DBG-based methods also introduce new issues. First, complex genomic variants and repeats may result in complicated graphs that are difficult to parse [17]. Second, short contigs may be discarded at the post-processing step, where branch pruning may cause many false negatives. Furthermore, sequences assembled by k-mers without variants have little contribution if the purpose is detecting variants. Only reconstructing the active regions spanning the variants is more efficient than considering all k-mers [13]. Although it is possible to extend DBG-based methods to SV detection, the lack of sensitivity to local events makes these approaches less suitable for finding variants in ambiguous regions, such as repeats [18]. This motivates the need for a method to detect variants in arbitrary genome regions directly from DNA-seq data.

We present 2-kupl, a k-mer-based bioinformatics pipeline that compares matched case and control samples to discover case-specific variants. 2-kupl identifies sequence fragments (contigs) specific to the mutant dataset and their wild-type counterpart in the control dataset. This operation is done without relying on a reference genome. We compare the accuracy and CPU-requirements of 2-kupl with that of other variant calling software using both simulated and real DNA-seq datasets. We analyze the nature of novel variants detected by 2-kupl and potential reasons for their absence in conventional protocols. We also use 2-kupl to detect recurrent variants in prostate adenocarcinoma (PRAD) WES samples from the TCGA project [19]. Finally, we evaluate 2-kupl precision in bacterial WGS data. Overall, we demonstrate that 2-kupl is a practical and powerful alternative for the discovery of genomic variants in hard-to map regions or species with no reliable reference.

## Results

### A novel algorithm for detecting variants between two DNAseq samples

We developed 2-kupl to predict variants between pairs of matched DNAseq libraries. Input libraries consist of a “case” and a “control” sample such as a pair of tumor and normal tissues from one patient or a pair of mutant and wild-type bacterial strains. Data can be either WGS or WES. 2-kupl extracts case-specific k-mers (cs-kmers) and matching control k-mers (ct-kmers) corresponding to a putative mutant and reference sequences and merges them into contigs. As 2-kupl begins with a shortlist of cs-kmers, the number of k-mers considered from unaltered regions and non-specific variants is drastically reduced compared with DBG-based methods (see Methods). If a reference genome is provided, 2-kupl can also align contigs to the reference and generate genomic coordinates just like with mapping-based methods.

### Performance on simulated WES data

We first applied 2-kupl to the detection of somatic mutations in a simulated human cancer WES dataset containing a known number of spliked-in mutations and indels. We compared 2-kupl with three other software, including two mapping-free methods (DiscoSNP++ and Lancet) and the leading mapping-based pipeline GATK-MuTect2. Results are summarized in the first column of Table 1. The number of cs-kmers to process is reduced by nearly 20% after data cleaning by 2-kupl.

**Table 1:** Number of k-mers and contigs after applying 2-kupl on two matched libraries

	Simulated WES	TCGA-ZG-A9ND WES
All k-mers (tumor/normal)	465,718,268/465,610,133	184,233,006/177,517,776
Raw cs-kmers	23599	393525
Cleaned cs-kmers	18439	291350
Matched cs-kmers	16914	240360
All contigs	1245	106426
Mutations	1026	9901
Indels	112	1105
Unmapped	0	58
Low confidence	107	312

88.6% of cs-kmers were matched to ct-kmer, corresponding to predicted point mutations or indels. We evaluated mutations and indel calls by 2-kupl and concurrent methods (Table 2). For mutation calling, 2-kupl performed better than the other mapping-free methods in terms of F1 score (Table 2). Lancet and GATK achieved better recall than 2-kupl, but Lancet also introduced more false positives. 2-kupl had a higher recall for calling indels than DiscoSNP++ and Lancet but was outperformed by DiscoSNP++ in FDR and precision (Table 3). Expectedly, GATK-MuTect2 outperformed all mapping-free approaches regardless of variant types. DiscoSNP++ did not perform as well as others in terms of recall ratio due to the different usage. DiscoSNP++ first pooled together two samples and screened case-specific variants afterwards. This procedure contributes to eliminate many false positives but also leads to ignoring some low frequency variants exclusively present in the case sample. Lancet performed well in terms of recall but at a high cost of false positives. As expected, most false positives had few reads containing the alternative allele, which is frequent with Lancet. The high recall and high rate of false positives produced by Lancet are consistent with the conclusions of Meng and Chen [20]. The GATK-MuTect2 pipeline outperformed all mapping-free approaches when calling mutations. The use of a reference sequence and the Haplotype Caller algorithm gives GATK-MuTect2 a clear advantage. Even though 2-kupl got a relatively lower recall than GATK-MuTect2, it had better control of the false positives and got a higher precision when calling indels (Table 3).

**Table 2:** Comparison of four approaches on mutations using simulated WES data

Mutations	2-kupl	DiscoSNP++	Lancet	GATK-MuTect2
True positive	581	373	604	689
False positive	45	3	126	2
False negative	241	530	218	133
Recall	0.71	0.41	0.73	0.84
FDR	0.07	0.01	0.17	0.003
Precision	0.93	0.99	0.83	0.997
F1 score	0.80	0.58	0.78	0.91

**Fig. 1:**
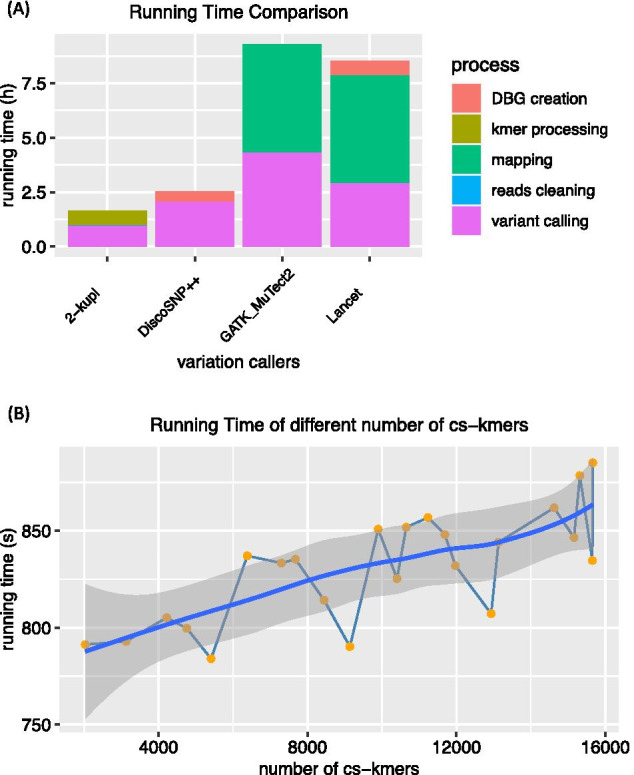
Running time and performance with different types of variants. **a** Overall running times of four software. The time consumed by each process in four protocols is marked in different colors. **b** Running times of 2-kupl for different numbers of cs-kmers. The line with dots represents the exact running time corresponding to certain number of cs-kmers. The solid line is the fitted line, and the shaded background is the confidence interval

Another advantage of 2-kupl is the short running time (Fig. 1a). 2-kupl took 1.6 h to analyze the simulated WES data with default parameters. DiscoSNP++ took 2.54 h to call variants from both case and control samples. Both Lancet and GATK-MuTect2 require prior mapping of reads to the human genome (which takes 3.17 h), explaining in part their longer runtimes.

To evaluate 2-kupl run time dependency on the number of cs-kmers, we ran 2-kupl on datasets with different numbers of cs-kmers (Fig. 1b). Running time increased linearly with the number of cs-kmers. Each additional 10,000 cs-kmers increased the running time by nearly 50 s.

**Fig. 2:**
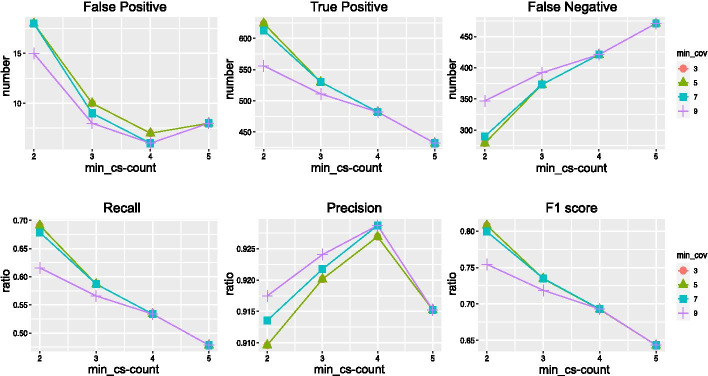
Robustness of 2-kupl using different parameters. The x-axis indicates the min_cs-count parameter and the y-axis represents the corresponding ratio or number. The thresholds of coverage and cs-count are denoted as min_cov and min_cs-count, respectively. The trend lines under different min_cov parameters are represented by four colors

We estimated the performance of 2-kupl under different parameter combinations. Coverage and cs-count thresholds (‘mim_cov’ and ‘min_cs-count’, respectively) were varied from 3 to 9. Results are shown in Fig. 2. The min_cs-count parameter was negatively related to recall and positively related to false negatives. The min_cov parameter was inversely related to F1 score, recall, FDR, and true positives. Precision reached an inflection point when min_cs-count was set to 4.

**Table 3:** Comparison of four approaches on indels using simulated WES data

indels	2-kupl	DiscoSNP++	Lancet	GATK-MuTect2
True positive	42	29	40	49
False positive	16	1	44	26
False negative	39	52	41	32
Recall	0.52	0.36	0.49	0.60
FDR	0.27	0.03	0.52	0.35
Precision	0.72	0.97	0.47	0.65
F1 score	0.60	0.52	0.48	0.63

**Fig. 3:**
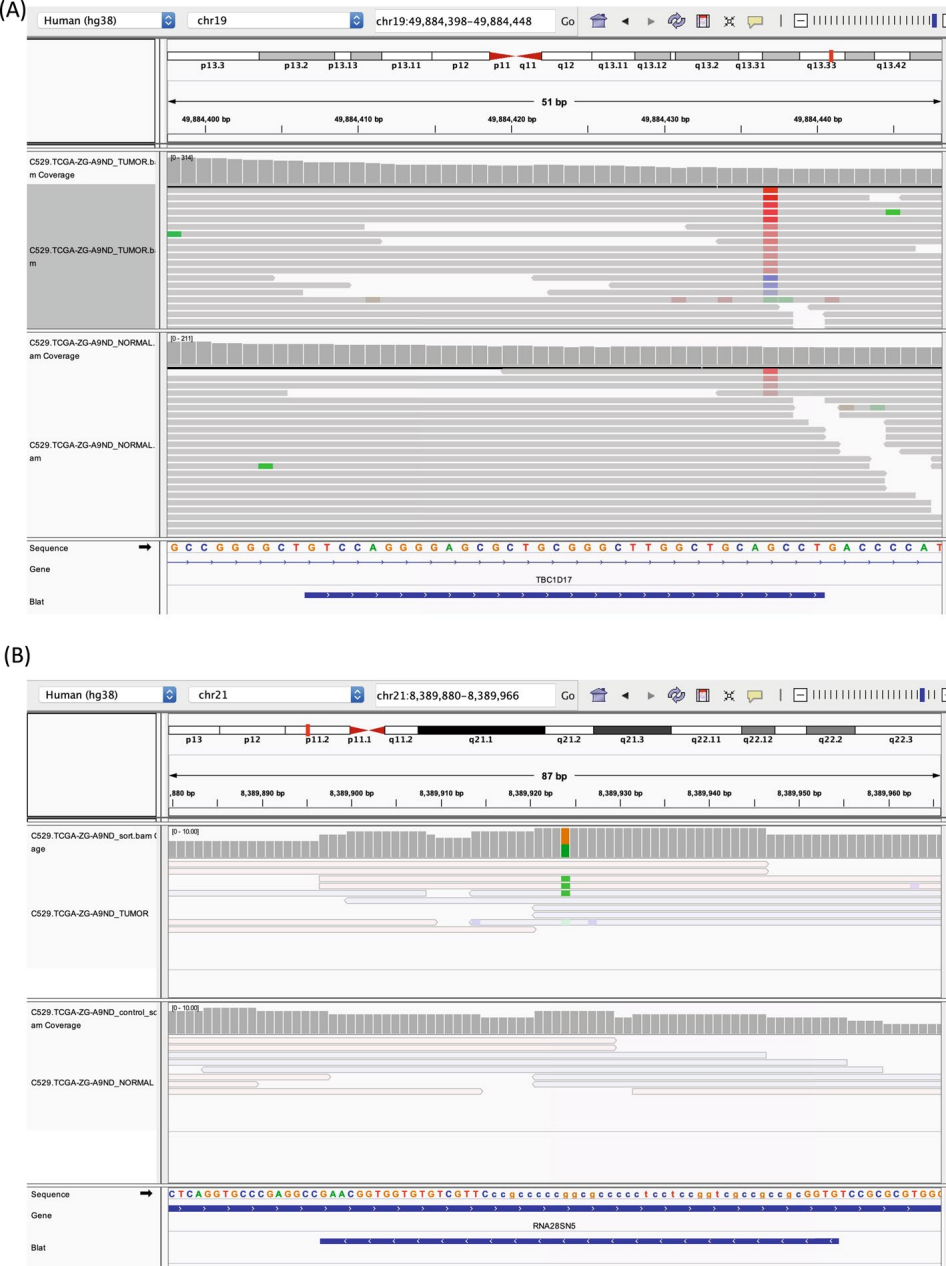
IGV views of variant calls in TCGA-PRAD WES dataset. The two central tracks show aligned reads from the tumor (top) and normal (bottom) WES library. The lower track shows gene annotation and 2-kupl contigs. **a** A likely false-positive call by 2-kupl at a position of low mapping quality, **b** A likely true positive within a repeat region. Reads in transparent color have low MAPQ (mapping quality) values (<10)

### Performance on simulated WGS data

We further benchmarked 2-kupl on a simulated WGS dataset with an average read depth of 50X (vs. 230 in WES). For mutation calls, 2-kupl and GATK-MuTect2 achieved the same recall ratio of 0.86 (Table 4). The precision of 2-kupl was slightly lower than GATK-MuTect2 but still above 0.9. For indels, the recall of 2-kupl dropped to 0.82 (Table 5). The false positive call rates of 2-kupl increased with WGS data relative to WES data due to the lower coverage of WGS. A limitation of 2-kupl is that false signals can not be ruled out by allele frequency in low coverage regions. Also, k-mers may be incorrectly considered as cs-kmers when there is not enough reads covering the locus in the control sample.

**Table 4:** Comparison of 2-kupl and GATK-MuTect2 on mutations using simulated WGS data

mutations	2-kupl	GATK-MuTect2
True positive	13835	13920
False positive	1248	30
False negative	2220	2135
Recall	0.86	0.86
FDR	0.08	0.002
Precision	0.91	0.99
F1 score	0.89	0.93

**Table 5:** Comparison of 2-kupl and GATK-MuTect2 on indels using simulated WGS data

indels	2-kupl	GATK-MuTect2
True positive	3315	3620
False positive	504	108
False negative	750	445
Recall	0.82	0.89
FDR	0.13	0.02
Precision	0.84	0.96
F1 score	0.84	0.92

The simulated WGS dataset contained 157 SVs (deletions, duplications, and translocations longer than 50bp). Expectedly, GATK-MuTect failed to detect the majority of SVs (Table 6). We thus compared 2-kupl with Delly, a software that finds structural variants based on aligned reads [21]. Overall 2-kupl had a slightly lower precision and recall than Delly (Table 6). We investigated 22 SVs missed by Delly and captured by 2-kupl. We found these reads were left unmapped by BWA due to multiple hits in the genome and thus could not be assessed by Delly (Additional file 6: Table S5). An advantage of 2-kupl here is that all k-mers covering SV junctions are kept and assembled regardless of mapping status. Furthermore, 2-kupl is capable of detecting small variants in the same run.

**Table 6:** Comparison of 2-kupl, GATK-MuTect2 and Delly on structural variants using simulated WGS data

mutations	2-kupl	GATK-MuTect2	Delly
True positive	133	49	135
False positive	27	0	16
False negative	24	108	22
Recall	0.85	0.3	0.86
FDR	0.17	0	0.11
Precision	0.83	1	0.89
F1 score	0.84	0.47	0.88

### Assessing 2-kupl on a real normal-tumor WES dataset

To assess 2-kupl results on actual WES data, we applied 2-kupl on one WES dataset of matched tumor and normal tissues from the TCGA-PRAD dataset. We first compared 2-kupl and GDC portal somatic variant calls (see Methods) on the TCGA patient with the highest tumor mutational burden. The numbers of k-mers, contigs and variants obtained by 2-kupl are shown in the second column of Table 1. Mutation calls by 2-kupl and GDC portal variants are shown in Table 7. Although total call numbers were similar, only 327 calls ( 9%) were shared by the two approaches, including 319 mutations and 8 indels. Among the variants detected by 2-kupl, 193 (5.13%) mapped to noncoding regions and 101 (2.7%) were annotated as repeats by RepeatMasker [22]. 2-kupl also captured 57 (1.5%) unmapped variants. 173 2-kupl variants (4.6%) were mapped to low mappability “blacklist” regions [23]. In spite of the small general overlap of 2-kupl and GDC portal variants, the two methods have a much stronger agreement on high scoring 2-kupl calls (Additional file 1: Fig. S1A). Of note, mutation calls obtained on the same sample by four different mapping-based protocols also show poor consistency (Additional file 1: Fig. S1B).

**Table 7:** Number of mutations and indels detected by 2-kupl and GDC portal variants

	2-kupl	GDC portal variants	overlap
Mutation	3607	3093	319
Indel	151	823	8
Total	3758	3916	327

We further analyzed mutations specific to 2-kupl. These calls may have been rejected in GDC portal variants for a number of valid reasons, including low mapping quality, location in short tandem repeats or presence in normal samples. A real “miss” by the reference-based pipeline should be recorded only when reads could not possibly be aligned to the genome while they indeed contained a valid mutation.

Figure 3a shows a case of false positives introduced due to artifactual cs-kmers. Generally, k-mers harboring a mutation present in both tumor and normal tissues are supposed to be ruled out. However, erroneous tumor-specific “cs-kmers” can escape the filtering process if the same k-mer in the normal tissue happens to be low quality and is discarded.

**Fig. 4:**
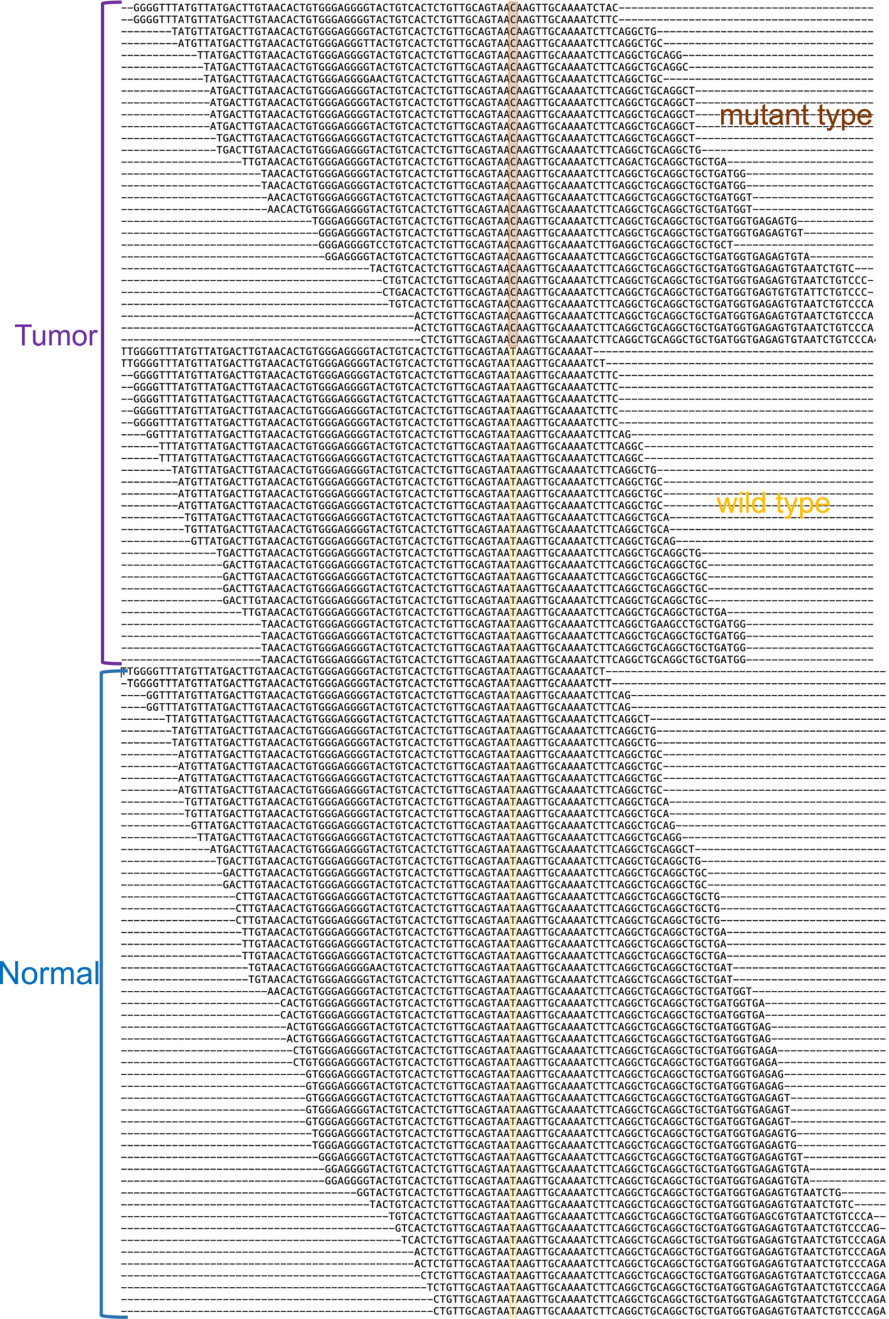
An unmapped somatic variant from a TCGA PRAD patient. Only reads matching the central k-mer of the tumor-specific variant or its inferred counterpart are shown. Reads from the tumor and normal samples are distinguished. The position of variation is highlighted

Certain 2-kupl specific mutations are possibly true positives discarded by mapping-based protocols due to their location within a repeat region. Figure 3b shows such a potential somatic mutation. The mutation is located within a ribosomal RNA gene that is repeated multiple times in the genome and further contains a C-rich repeat (represented in lower cases). Reads generated from these repetitive regions are given low MAPQ values by mappers and variants in these regions are then discarded by variant callers.

Among unmapped 2-kupl calls, only one has a Phred score in the top 5% (Additional file 1: Fig. S2). The mutant sequence and its inferred reference are shown in Additional file 1: Fig. S3. The mutant contig is covered by 0 and 47 reads in the Normal and Tumor sample, respectively while the reference is covered by 88 and 65 reads in the Normal and Tumor sample, respectively (Fig. 4). The sequence maps to a centromeric repeat of Chr22, with three mismatches. The mapping procedure would thus miss this highly significant variant.

### Recurrent mutations in TCGA-PRAD

Recurrence across patients is a powerful criterion for distinguishing drivers from passenger mutations [24–26] and has been used to discover drivers and define molecular subtypes of prostate cancer [27]. We applied 2-kupl to each pair of Normal/Tumor samples in the complete PRAD WES dataset (N=498) and identified 3211 recurrent variants (Additional file 2: Table S1). For comparison we retrieved from the GDC portal recurrent variants predicted for the same dataset (GATK-MuTect2 pipeline, see Methods). Among 3734 recurrent variants in the GDC portal, 854 were shared with 2-kupl recurrent variants (Additional file 2: Table S1). We further compared the recurrent variants to a comprehensive dataset of recurrent prostate cancer mutations from Fraser et al. [28] based on 200 whole-genome and 277 whole-exome sequences from multiple sources. Comparisons were restricted to exonic regions. Within the 48 recurrent mutations in exonic regions from Fraser et al, a similar number was shared with 2-kupl or the GDC-portal (22 and 21, respectively) (Additional file 3: Table S2). Among recurrent mutations specific to 2-kupl, we note the one found at chr14:37592023 within an exon of FOXA1, a putative prostate cancer driver [29], in three TCGA-PRAD patients.

**Fig. 5:**
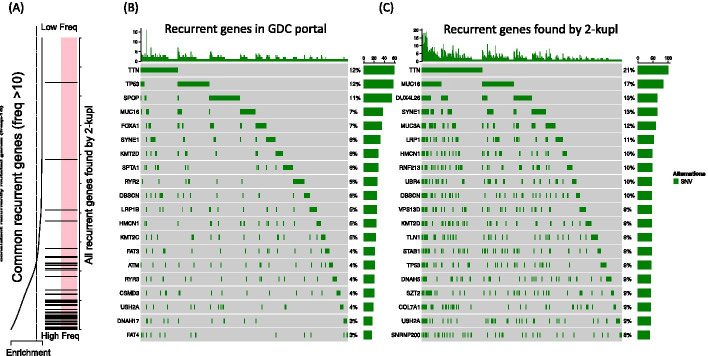
Recurrently mutated genes in the TCGA-PRAD WES dataset. **a** Enrichment analysis of recurrent genes. The vertical bars are the common recurrently mutated genes (altered in at least ten patients) between GDC portal and 2-kupl. The x axis represents the recurrent genes found by 2-kupl sorted by frequency. The smooth curve reflects the degree to which the common genes are overrepresented in the whole 2-kupl recurrent genes. **b** The 20 genes with the highest mutational frequency detected in GDC portal variants. **c** The top 20 recurrent genes with the highest mutational frequency detected by 2-kupl

We further compared 2-kupl calls to GDC portal variants at the level of genes (Detailed in Method section). The GDC portal reported 6944 genes mutated in two or more patients, versus 14137 recurrent genes by 2-kupl. Enrichment analysis shows a good convergence of the most frequently mutated genes by the two methods (Fig. 5). Figure 5b, c show oncoplot views of the top 20 genes according to the GDC portal and 2-kupl, respectively, showing eight shared genes. Both gene lists are contaminated by long (TTN) or highly polymorphic genes (Mucins) whose recurrence is an artifact due to higher mutation counts. Although many software are available to account for those effects [30], we purposely analyze the uncorrected list of genes here. Among the top 20 mutated genes by 2-kupl and GDC portal, 7 and 9 genes, respectively, are known prostate cancer-related genes. Among those, UBR4, DNAH5 and LRP1 were only detected by 2-kupl. When considering the top 50 recurrently mutated genes according to 2-kupl and GDC portal, 19 and 23, respectively, are cancer-related. Among those, HSPG2, DNAH3, UBR4, COL6A3, CABIN1, IGF2R, PTPRF, DNAH5, HTT and TRRAP were only detected by 2-kupl.

UBR4 contains 48 2-kupl mutations, more than any other gene. Additional file 1: Figure S4 shows read alignment at this gene for patient TCGA-EJ-7125 who carries the most UBR4 mutations (8/48 mutations). While seven of these mutations are absent in GDC portal variants, all can be visually validated as tumor-specific mutations as per the IGV display (Additional file 1: Fig. S4 A-G).

**Fig. 6:**
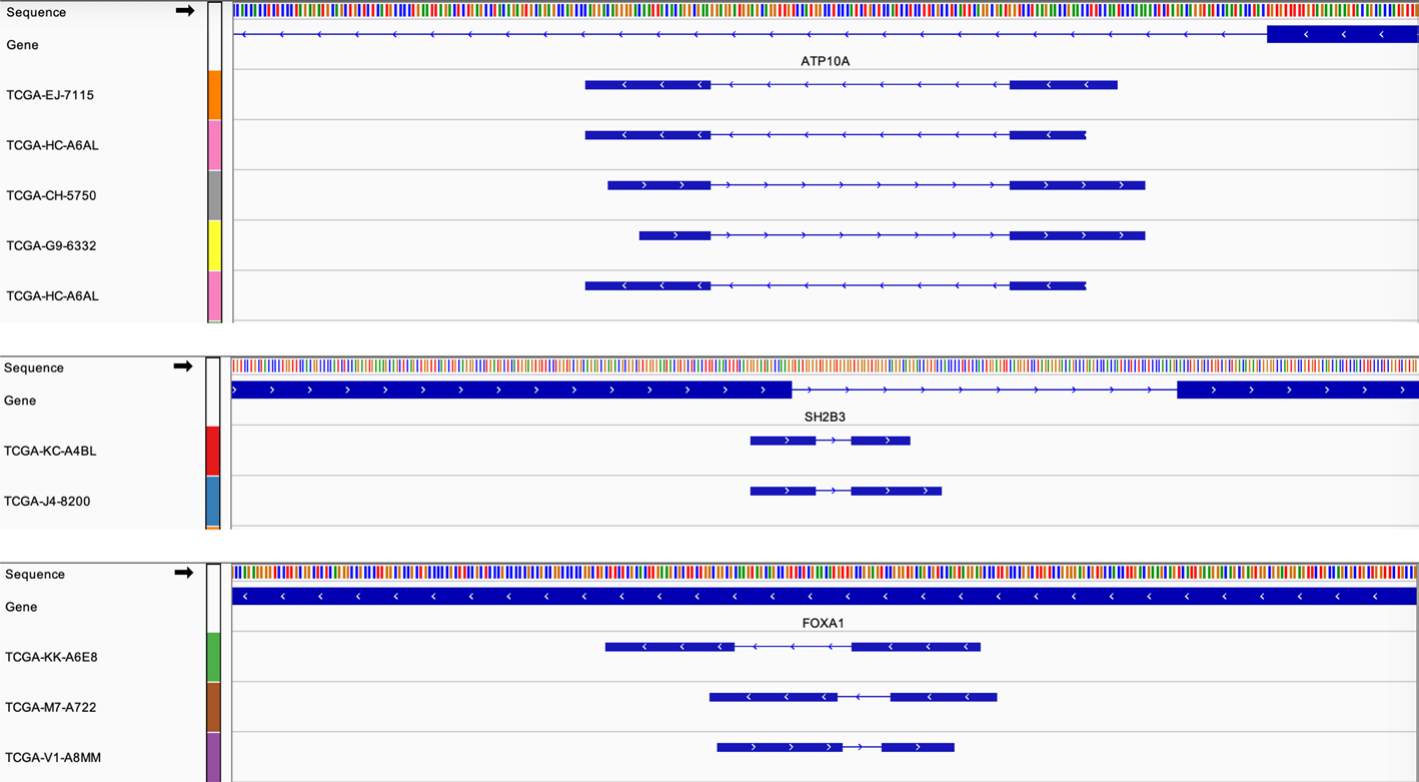
Recurrent structural variants mapping to three prostate cancer genes. In each track, lines represent the genome sequence (top), annotated genes, and variant contigs identified in different patients

Besides recurrent mutations and indels, we found 20 genes with 43 recurrent structural variants predicted in at least two patients (Additional file 2: Table S1). All these predicted variants can be supported by at least one read from the tumor library. Three recurrent structural variants map to prostate cancer genes SH2B3, ATP10A and FOXA1 (Fig. 6). Variants in gene ATP10A and SH2B3 have exactly the same junctions in at least two patients. As the three variants in gene FOXA1 impact on the same exon, we grouped them as one same recurrent event despite not representing the exact same variation. All these recurrent structural variants are longer than 10bp. State-of-the-art procedures usually miss such variants at the mapping stage.

### Performance on bacterial WGS data

2-kupl can be applied to pairwise comparisons of DNA-seq datasets in any species. We present here an application to bacterial whole genome sequences. A frequent problem in bacterial genetics is identifying mutations in strains for which no reliable reference genome is available. We investigated the performance of 2-kupl on 21 DNA-seq datasets from a *Pseudomonas aeruginosa* strain, in which 26 variants had been previously identified and confirmed by geneticists (see Methods).

**Fig. 7:**
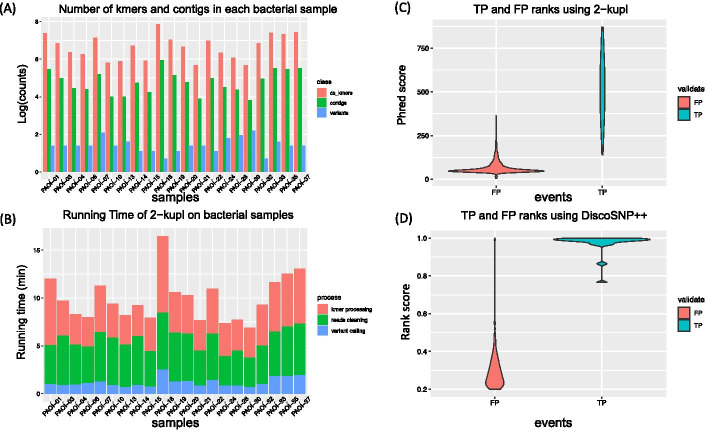
Performance of 2-kupl on bacterial DNA-seq datasets. **a** Number of cs-kmers, contigs and variants are shown for each bacterial sample. **b** Running time of 2-kupl on each sample is shown for different steps. **c** Distribution of Phred scores computed by 2-kupl in TP and FP events. **d** Distribution of DiscoSNP++ score ranks in TP and FP events

About 141 variant contigs were predicted on average for each pair of WT/mutant strains, with an average running time of 10 minutes (Fig. 7a, b). Score ranking by 2-kupl and DiscoSNP++ allowed a clear separation of TP from FP (Fig. 7c, d). True positive calls were ranked first in 19 out of 19 mutant samples by 2-kupl and in 16 out of 16 samples by DiscoSNP++. Compared with Phred scores used in 2-kupl, DiscoSNP++ scales the rank scores from zero to one and thus the true positive variants are more concentrated.

2-kupl could recall all true positive variants, including SNVs and large deletions longer than 100 bp, while DiscoSNP++ missed three large deletions (555 bp, 213 bp and 109 bp, Additional file 5: Table S4). Meanwhile, DiscoSNP++ obtained 129 false positives versus 45 for 2-kupl (Table 8). Therefore 2-kupl had the best recall and precision on this dataset, especially for large indels.

**Table 8:** comparison between 2-kupl and DiscoSNP++ on the bacteria DNA-seq data

	2-kupl	DiscoSNP++
True positive	26	23
False positive	45	129
False negative	0	3
Recall	1	0.88
FDR	0.64	0.85
Precision	0.36	0.15
F1 score	0.52	0.26

## Discussion

Most variant detection protocols rely on reference genomes. However, even for species with a high-quality reference genome such as humans, depending on a reference is subject to limitations. Genomes contain large numbers of highly variable, repetitive or otherwise unmappable regions, which are unsolvable by short-read sequencing techniques. Hundreds of unsolved regions remain in telomeres and centromeres, also known as ‘dark matter’ [31]. The X chromosome is the only complete human chromosome as of today [32]. Pathogenic variants within these unannotated regions are easily missed by mapping-based approaches due to low mapping quality, especially with low depth in whole-genome sequencing. Furthermore, the human genome varies across individuals and populations and a single reference genome does not account for this diversity [33].

2-kupl is able to detect variants, including mutations, indels and structural variants, without relying on a reference genome. Based on matched DNA-seq data, 2-kupl captures case-specific k-mers and counterpart k-mers (i.e. without the variation) into the same bucket. Sequence contigs harboring a local variation and its putative reference are inferred through the assembly of k-mers in each bucket.

To control artifacts induced by sequencing errors, 2-kupl takes both base quality and coverage into account. The general sequencing error rate in short-read NGS data is larger than 0.1% [34]. It is worth consuming computing resources and running time to remove these 0.1% artifacts because these sequencing errors result in large numbers of artifactual cs-kmers. To reduce the impact from low-quality bases, we combine Cutadapt and an ‘OverrideN’ function that flags low quality bases in the mid part of reads. This significantly reduces the number of cs-kmers and speeds up the computing procedure.

We compared the performance of 2-kupl with that of three competing methods in terms of running time, recall and precision. 2-kupl outperformed mapping-free methods DiscoSNP++ and Lancet in terms of recall or precision but did not reach the performance of the state-of-the-art alignment-based GATK-MuTect2 on human data.

DiscoSNP++ suffers from limitations of DBG data structures in regions with sequencing errors, genomic variants and repeats [18]. Efficient solutions searching for bubbles from such complicated structures are still under development. Furthermore, short contigs may be discarded within the post-process, cutting branches, for instance [35]. In our bacterial DNA-seq analysis, DiscoSNP++ missed three validated large deletions.

Lancet has a higher recall ratio than 2-kupl but also introduces more false positives. Furthermore, Lancet missed variants from repetitive regions and is not able to detect fusions from distant regions.

2-kupl has a higher F1 score than DiscoSNP++ and Lancet and performs better in terms of recall ratio or precision than either of them. Expectedly, 2-kupl did not outperform GATK-MuTect2 on WES data. First, GATK-MuTect2 uses a sophisticated Bayesian model to estimate a genotype’s likelihood given the observed sequence reads that cover the locus. When GATK-MuTect2 encounters a region showing signs of variation, it discards the existing mapping information and completely reassembles the reads in that region. This allows GATK-MuTect2 to be more accurate when calling regions that are traditionally difficult to call. Despite slightly fewer true positives, 2-kupl also detects fewer false positives than GATK-MuTect2. It is worth mentioning that 2-kupl has the lowest time complexity among the four methods.

By applying 2-kupl to the TCGA-PRAD patients, we were able to detect recurrent mutations and indels missed by the GDC portal’s GATK-MuTect2 pipeline. Reads in these regions have either low mapping qualities or multiple hits and were discarded in the GDC portal pipeline. Mapping-based methods all suffer from this issue and are powerless when faced with low complexity regions. 2-kupl identified recurrent mutations and recurrently mutated genes in high agreement with GATK-MuTect2. Mutated genes were enriched in PRAD-related genes, some of which specific to 2-kupl. As an example, we visually confirmed multiple 2-kupl-specific mutations in UBR4. Recurrent variants detected from the unmappable regions by 2-kupl provide insights into potential novel somatic variants even though the locus of origin of the contig sometimes cannot be determined.

Standard variant calling pipelines may miss mutations for multiple reasons: low allele frequencies, tumor contamination, ambiguities in short read alignment, inadequate sequencing depth, high GC content, sequencing errors and ambiguities in short read alignment. Different programs are affected by these factors to varying degrees. As a consequence, the mutations called by different pipelines are not consistent [36]. 2-kupl is not affected by some of these sources (GC content, alignment artifacts and mappability) and can detect a number of recurrent mutations (ie. potential driver events) that are not found by standard pipelines.

Several natural directions exist for extending 2-kupl. First, 2-kupl lacks sensitivity in detecting structural variants. All cs-kmers covering the junction are retained and extended to contigs. Unfortunately, neither the ct-kmers nor the reads are easily obtained when considering a hamming distance of one. A structural variation can be detected only if enough supporting reads are covering at least one side of the variation. Focusing on the cs-kmers regardless of ct-kmers could address this problem but at the cost of more false positives. A second limitation occurs when control samples are contaminated with tumor cells, which is relatively frequent in tissue biopsies. To address this problem, 2-kupl includes a parameter representing a k-mer count threshold in the control sample. However, a fixed contamination threshold may introduce unwanted non-specific variants. Future works should evaluate probabilistic approaches to address this issue.

## Conclusions

In conclusion, the identification of different kinds of variants, using DNA-seq data, remains challenging. The leading protocols developed for DNA-seq highly rely on the reference. In general, the methods that align sequencing data to the reference (mapping-based methods), perform better than do the mapping-free methods. However, 2-kupl can capture events falling into the difficult-to-map regions, and can perform better than other mapping-free protocols. 2-kupl is the fastest tool in the comparison with other methods because the mapping procedure is not included. The high agreement in top ranking variants by 2-kupl and GDC portal variants indicates the capacity of using 2-kupl as an extension and supplementation of the mapping-based methods.

## Methods

### Outline of 2-kupl pipeline

**Fig. 8:**
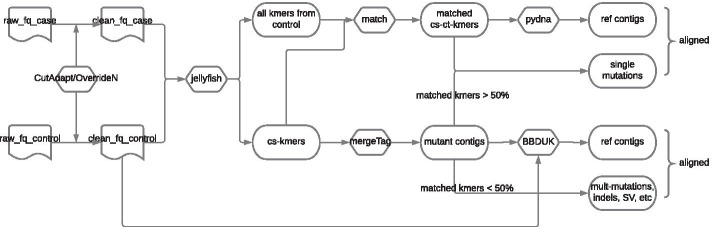
Overall workflow of 2-kupl. This flowchart describes the analysis process of 2-kupl, including the input and output file format and function of each module

The general pipeline is presented in Fig. 8. The input is composed of DNA-seq data from two matched samples. Samples typically correspond to control/normal/wild-type and a case/tumor/mutant-type. For cancer data, we strongly recommend using as a control of a distant tissue such as white blood cells rather than adjacent normal tissues, as the later can be contaminated by tumor cells and 2-kupl only considers variant sequences that are absent in the control dataset. Sequence types can be either single-end or paired-end sequencing reads. 2-kupl then identifies pairs of case-specific k-mers (cs-kmers) and counterpart k-mers (ct-kmers). 2-kupl returns predicted variants exclusive to the case sample, including mutations, indels and structural variations. Variant statistics including cs-count, coverage, allele frequency and variant P-value are computed. A variant file and an alignment file are produced. 2-kupl accepts multiple threads and uses 10 threads by default.

2-kupl is developed purely in Python. The main dependencies include Jellyfish [37] and GSNAP [38]. Other dependent python libraries and instructions can be found from the Github repository https://github.com/yunfengwang0317/2-kupl

### Data cleaning

Low quality sequences are trimmed with Cutadapt [39] (parameter ‘–quality-cutoff’ = 10). As Cutadapt does not remove low-quality bases within the central part of reads, we implemented an overriding function that replaces each low-quality base (Phred score<10) with N. This procedure is applied to both case and control libraries.

### k-mer indexing and counting

Jellyfish is used to index and quantify k-mers from both case and control with options k=31 and -C (canonical k-mers). As Jellyfish removes k-mers containing Ns, none of the low-quality bases is present in the k-mer list. The generated k-mers subsequently undergo two filtering steps. First, k-mers with counts below a user-specified cutoff (default=3) are removed. These low abundance k-mers are assumed to result from sequencing errors or off-target regions in the case of WES data. Second, k-mer lists from case and control are compared and only case-specific k-mers (cs-kmers) are retained.

### Matching counterparts of cs-kmers

**Fig. 9:**
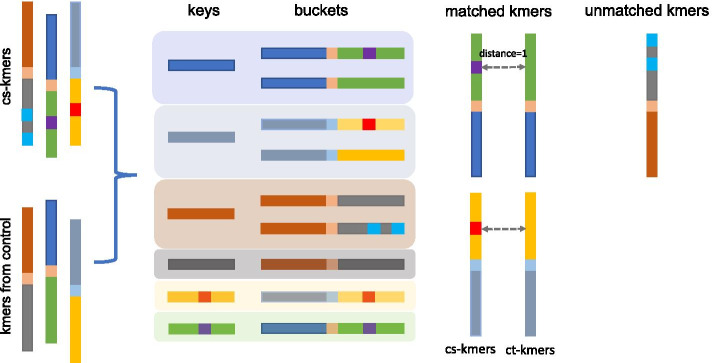
Procedure for matching cs-kmers to ct-kmers. Long rectangles represent one 31-mer. Short rectangles (keys) represent the head or tail 15 bp of a cs-kmer. Color changes indicate sequence differences

For each cs-kmer harboring a point mutation, there should exist a counterpart k-mer (ct-kmer) from the control dataset with only one base substitution (Hamming distance =1), which can be considered as a product of the wild type sequence. Note that Hamming distance=1 only considers substitutions. Hence single nucleotide insertions and deletions are rejected at this step and will be treated later with unmatched k-mers. Finding the matched ct-kmer for each cs-kmer should allow us to infer the variation without reference sequences. We initially build a hash table where the keys are the continuous 15 bases from each side of cs-kmers. For each 15-bases key, we create a bucket of all k-mers starting or ending with the key. Then we survey the buckets and seek all k-mer pairs with a hamming distance of one in the same bucket. We thus generate all k-mer pairs (ki, kj) with a hamming distance of one. For any pair of k-mers with a Hamming distance of one, if one k-mer comes from the cs-kmer list and the other comes from the control, this pair of k-mers is considered to be matched. Otherwise, we allocate the cs-kmers to the “unmatched k-mers” group. These unmatched k-mers either contain variants of more than one nucleotide (multiple mutations, indels and structural variants) or come from low coverage regions. The schematic workflow is shown in Fig. 9.

### Assembly of cs-kmers into mutant contigs

cs-kmers are assembled into mutant contigs that correspond to variants and their local context. The assembly process is done using the “mergeTag” function from DEkupl [40] (https://github.com/Transipedia/dekupl). Two k-mers overlapping by k-i bases are merged iteratively with i ranging from 30 to 25 (min_overlap parameter is set to 25 by default). The merging process is interrupted when no k-mers can be added or ambiguity occurs (two different overlapping k-mers are encountered).

### Inferring reference contigs

We use two distinct procedures for reference sequence determination, depending on whether or not sufficient ct-kmers are available to build a reference contig.

For each mutant contig, if more than half of its component k-mers are matched, all the ct-kmers are merged by the python package pydna [41]. The resulting mutant contigs correspond to isolated mutations. Merged contigs produced by ct-kmers can be regarded as putative references. For each pair of mutant and reference contig, we then define two values representing counts of supporting k-mers for the mutant allele (cs-count) and supporting k-mers for both mutant and reference alleles (coverage). The cs-count is computed from the median k-mer count of cs-kmers and coverage is calculated from the sum of the median count of cs-kmers and ct-kmers. Herein, we select the median count instead of the mean count because mean values are more sensitive to high-count k-mers from repeats or copy number amplification regions.

For mutant contigs in which less than half of the k-mers are paired, we consider that a reference cannot be assembled from paired-kmers. A procedure was implemented to retrieve the reference from the original reads. Reads with at most one mismatch to any k-mer from the mutant contig are retrieved from the control fastq file using BBDUK [42]. These reads are then assembled by CAP3 [43]. In this way, we can infer the putative reference for each contig and evaluate coverage based on the number of reads retrieved by BBDUK. The cs-kmers in these contigs have no matching ct-kmers and contigs are thus considered to contain multiple mutations, indels and structural variants (Additional file 6: Table S5).

### Filtering low-quality variants

The cs-count and coverage substantially impact the reliability of events called by 2-kupl. For instance, a sequencing error could be repeatedly generated in a region of high coverage. Besides, sequencing errors may, by chance, be detected as mutations with high allele frequency in low coverage regions. Thus, false positives are introduced due to either high cs-count in high coverage regions or high allele frequency in low coverage regions. However, coverage varies between whole-genome sequencing (WGS) and whole-exome sequencing (WES) data. WGS does not use an upfront enrichment step so it generates a more uniform coverage of the genome. On the other hand, the enrichment steps involved in WES lead to non-uniform coverage, generating coverage ‘hot’ and ‘cold’ spots [44]. 2-kupl provides several criteria for users to evaluate call reliability. A Fisher’s exact test P-value is calculated based on the cs-count and coverage in case and matched control libraries for each variation. A Phred quality score is subsequently computed as $-10log_{10}$P. Users can specify cutoffs for cs-count, coverage, allele frequency and Phred to filter false positives. Default cutoffs for cs-count, coverage, allele frequency and Phred are set to 3, 10, 0.05 and 5, respectively.

### VCF format export

Events identified by 2-kupl are exported as a variant call format (VCF) file [45]. 2-kupl outputs the contig harboring the variation and the corresponding putative reference without the variation for each event. If users provide an available reference, the mutant contig is mapped to this reference using GSNAP [38]. After the mapping process, actual chromosome and position information are provided in the VCF file. Besides the VCF file, 2-kupl also exports an alignment of each contig and its putative reference obtained using the pairwise2 python package [46]. Contigs corresponding to indels and structural variants are further mapped to reference by BLAST [47] (default parameters) which we found better suited to fragmented alignments.

### Comparison with other software

DiscoSNP++ [15] is designed for detecting SNVs and small indels from fastq files without using reference. DiscoSNP++ first generates a DBG of two matched samples pooled together [48] and detects variants based on searching bubbles in the graph. The context contigs can be extracted from DBG bubbles that correspond to local variants. As DiscoSNP++ calls variants in each sample rather than specific to one sample, we applied cutoffs to DiscoSNP++ allele frequencies (AF) to extract case-specific calls as found by 2-kupl. After testing multiple combinations, DiscoSNP++ achieved the best performance when AF cutoffs for both case and control samples were set to 0.05. Lancet [16] relies on localized colored DBG to detect somatic variants in paired samples. K-mers shared by two matched samples or specific to either of them are marked in different colors in the DBG. In this way, Lancet is able to detect case-specific events. It is worth mentioning that Lancet uses bam format files as input so it also leverages the reference before variant detection. We also compared 2-kupl with the leading reference-based GATK-MuTect2 pipeline [7]. GATK-MuTect2 takes mapped sequence files as input, detects variants based on the reference and compares the variants of two matched samples to identify case-specific variants (somatic mode). Version hg38 of the human genome was used in all reference-based procedures. To make runtime comparisons fair, we took the mapping procedure into account in Lancet and GATK-MuTect2. Alignment was performed using BWA with default parameters. Thus all four protocols started with fastq files. To evaluate the dependency of 2-kupl running time on the number of k-mers, we ignored the part up to k-mer counting. Mapped reads were visualized with the Integrative Genomics Viewer (IGV) [49] 2.6.2 on hg38. For structural variant detection in simulated WGS data, we also compared 2-kupl with Delly [21] a structural variant discovery software. Delly uses BAM alignment files as input and infers structural variants at single nucleotide breakpoint resolution using both insert size and split reads information.

### Simulated WES analysis

We downloaded simulated WES data from Meng and Chen [20]. This dataset was developed based on the NA12878 pilot genome [50] (reference data set of 5.4 million phased human variants validated by genetic inheritance from sequencing a three-generation 17-member pedigree). The authors used BAM-Surgeon [51] to select genomic loci and introduce random SNV and indel spike-ins, and generated 2x100nt reads WES files at 230X coverage. For our benchmark, we used a tumor sample described by authors as one of the most complicated, NA12878_79_snv_indel_sorted.bam (with four sub-populations, expected variant allele frequency (VAFs) of 0.5, 0.35, 0.2 and 0.1). Picard was used to convert bam files to fastq format files with default parameters. 2-kupl was run using default parameters on pairs of simulated normal-tumor fastq files.

### Simulated WGS analysis

A simulated WGS dataset containing two matched samples was generated by DWGSM (https://github.com/nh13/DWGSIM), with a mean coverage of 50X across available positions. The rates of mutations in case and control group samples were set as 0.0001 and 0, respectively. The fraction of indels in all variants was restricted to 20%. The expected VAF ranged from 0.1 to 0.5. All other parameters were set as default values. Besides the mutations and indels, the simulated WGS dataset also included structural variants including deletions, duplications and translocations longer than 50 bp. DWGSM generates fastq format files that are directly used as input for 2-kupl.

### TCGA-PRAD data analysis

Matched normal-tumor WES data of 498 patients from TCGA-PRAD (Prostate Adenocarcinoma) [52] were retrieved with permission from dbGAP [53]. BAM files were converted to paired-ends fastq files using Picard tools with default parameters. 2-kupl somatic variant calls were obtained for each normal/tumor pair using default parameters. Detailed analysis of variant calling was performed on the TCGA-PRAD sample with the highest tumor mutational burden (barcode TCGA-ZG-A9ND).

2-kupl results on the TCGA-PRAD dataset were compared to variant calls downloaded from the GDC portal. Briefly, the GDC portal workflow uses BWA to map reads to the human genome and determines variants with five state of the art variant callers, as described here: https://docs.gdc.cancer.gov/Data/Bioinformatics_Pipelines/. We used the maftools R package [54] to retrieve variants predicted using the GATK-MuTect2 pipeline and filtered against a “panel of normals”. This mutation dataset is hereafter referred to as the “GDC portal” dataset.

To remove putative germline variants from 2-kupl results, we built a boolean matrix representing the presence of each k-mer in each normal sample. Any k-mer present in at least two normal samples was excluded. Retained recurrent variants were considered as tumor-specific (Additional file 2: Table S1). Mutations detected by 2-kupl and absent in the GDC portal variants were considered as 2-kupl specific. To verify whether calls absent in GDC portal variants were not discarded at earlier stages of the GDC portal pipeline, we also retrieved the protected MAF file containing all unfiltered variants called by the MuTect2 workflow.

The oncoplot graph for GDC portal variants (Fig. 5a) was drawn using maftools. To obtain recurrently mutated genes by 2-kupl, we aggregated variants belonging to the same gene in 2-kupl results and constructed a gene-level occurrence matrix that was fed to maftools (Fig. 5b). Recurrent variants from 2-kupl and the GDC Portal were also compared with a comprehensive prostate cancer dataset from 200 whole-genome sequences and 277 whole-exome sequences from localized prostate tumours [28] (Additional file 3: Table S2)

Recurrently mutated genes were annotated using a collection of 1404 PRAD-related genes collected from CLINVAR [55], COSMIC [56], DISEASE [57], KEGG [58], OMIM [59], PheGenl [60] and driver predictions by Martincorena et al. and Armenia et al. [29, 61] (Additional file 4: Table S3).

### Bacterial genome analysis

We obtained WGS fastq files from the *Pseudomonas aeruginosa* PAO1Or wild-type strain and 24 phage-tolerant mutants [62]. Mutations in the phage-tolerant variants were previously validated by mapping of the WGS raw sequences to the PAO1Or genome (Genbank accession LN871187) and confirmed by PCR amplification and Sanger sequencing. We used one control WGS file and 21 mutant WGS files corresponding to 26 validated variants. Detailed variants (Additional file 5: Table S4) include seven mutations, 13 small indels and six large deletions longer than 100 bp. 2-kupl was run using default parameters on every mutant WGS file compared to the control WGS file.

## Supplementary Information

**Additional file 1: Fig. S1**. The distribution of shared SNVs in 2kupl and consistency of four mapping-based protocols. **Figure S2**. Phred score distribution. **Figure S3**. Alignment of the mutant contig and inferred reference from one unmapped event. **Figure S4**. IGV views of UBR4 mutations occurred on patient of TCGA-EJ-7125

**Additional file 2: Table S1**. This supplementary table includes recurrent SNVs, SVs and mutated genes identified by 2-kupl.

**Additional file 3: Table S2**. Comparison with the Fraser et al's recurrent PRAD mutations.

**Additional file 4: Table S3**. Prostate cancer related genes collected from various resources.

**Additional file 5: Table S4**. True positive variants in the bacterial WGS data.

**Additional file 6: Table S5**. 2-kupl detected structural variants that are missed by Delly.

## Data Availability

2-kupl is open source under MIT license and available at GitHub https://github.com/yunfengwang0317/2-kupl.

## Abbreviations

WES: Whole-exome sequencing
WGS: Whole-genome sequencing
TCGA: The Cancer Genome Atlas
PRAD: Prostate Adenocarcinoma
GDC: Genomic Data Commons
DBG: De Bruijn graph
MAPQ: Mapping quality
SV: Structural variant
