# Supplementary material for: 2-kupl: mapping-free variant detection from DNA-seq data of matched samples
Source: BMC Bioinformatics. 2021 Jun 5;22:304. doi: 10.1186/s12859-021-04185-6 (PMC8180056; doi:10.1186/s12859-021-04185-6)
Supplement: Supplementary file 1 — Additional file 1: Fig. S1. The distribution of shared SNVs in 2kupl and consistency of four mapping-based protocols. Figure S2. Phred score distribution. Figure S3. Alignment of the mutant contig and inferred reference from one unmapped event. Figure S4. IGV views of UBR4 mutations occurred on patient of TCGA-EJ-7125 [file 12859_2021_4185_MOESM1_ESM.pdf]

2-kupl: mapping-free variant detection from DNA-seq data of matched samples

Yunfeng WANG[1,2], Haoliang XUE[1], Christine POURCEL[1], Yang DU[2], Daniel GAUTHERET[1,3]

1. Institute of Integrative Cell Biology (I2BC), Université Paris-Saclay, CNRS, CEA, 1 avenue de la Terrasse, 91190 Gif-sur-Yvette, France
2. Annoroad Gene Technology Co., Ltd, Beijing, China
3. IHU PRISM, Gustave Roussy, 114 rue Edouard Vaillant 94800 Villejuif, France

Supplementary Figures and Legends

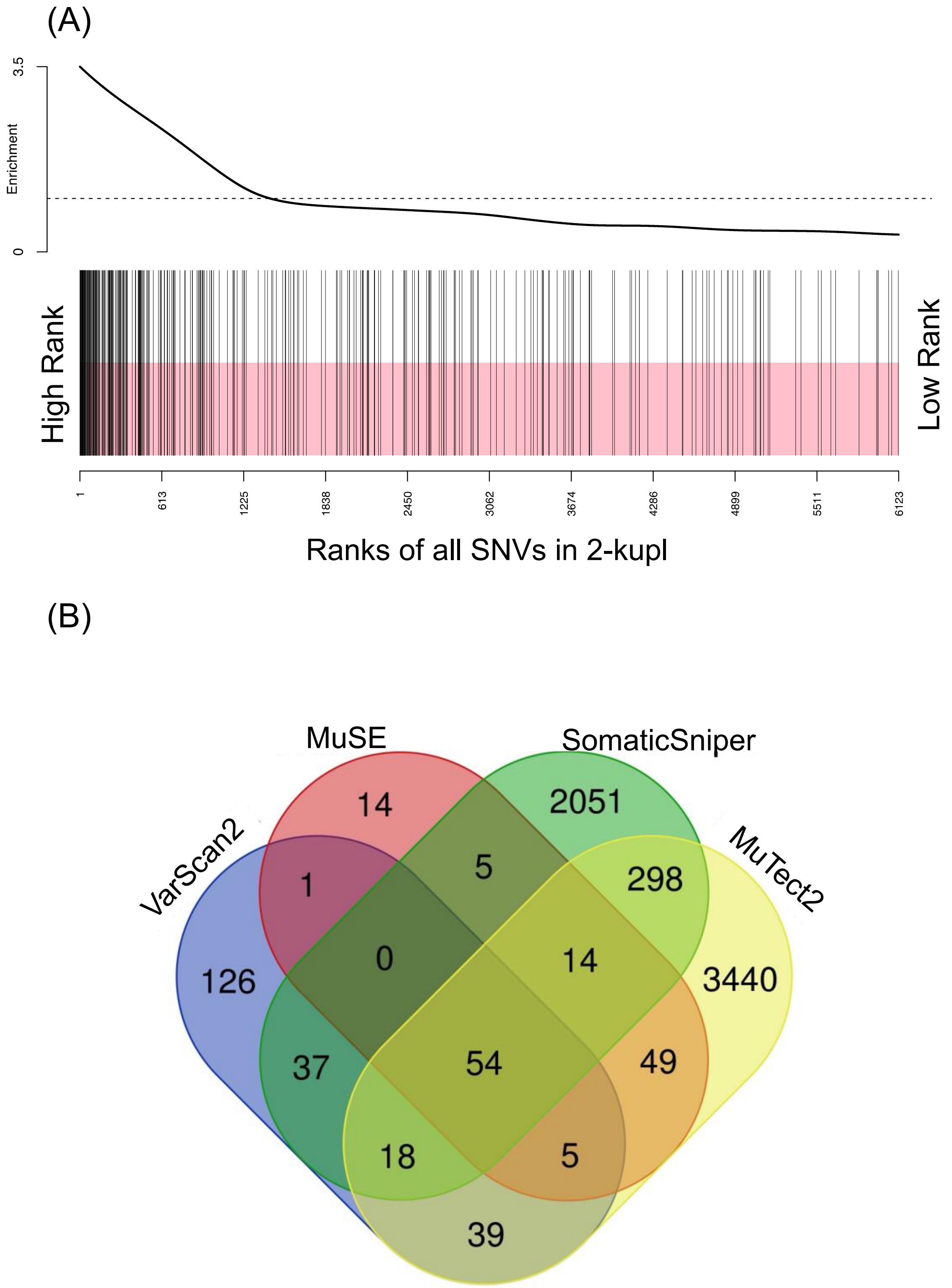

***Figure S1. Comparison of 2-kupl SNV calls with other software**. (A)Distribution of shared MuTect2/2-kupl SNV calls. The x axis represents 2-kupl SNV calls sorted by Phred score. Vertical bars are SNVs consistent between 2-kupl and MuTect2. The above curve reflects the degree to which shared SNVs are overrepresented among 2-kupl calls. (B) Venn diagram showing variant calls from each of the four callers retrieved from GDC portal. All the results correspond to the same PRAD patient.*

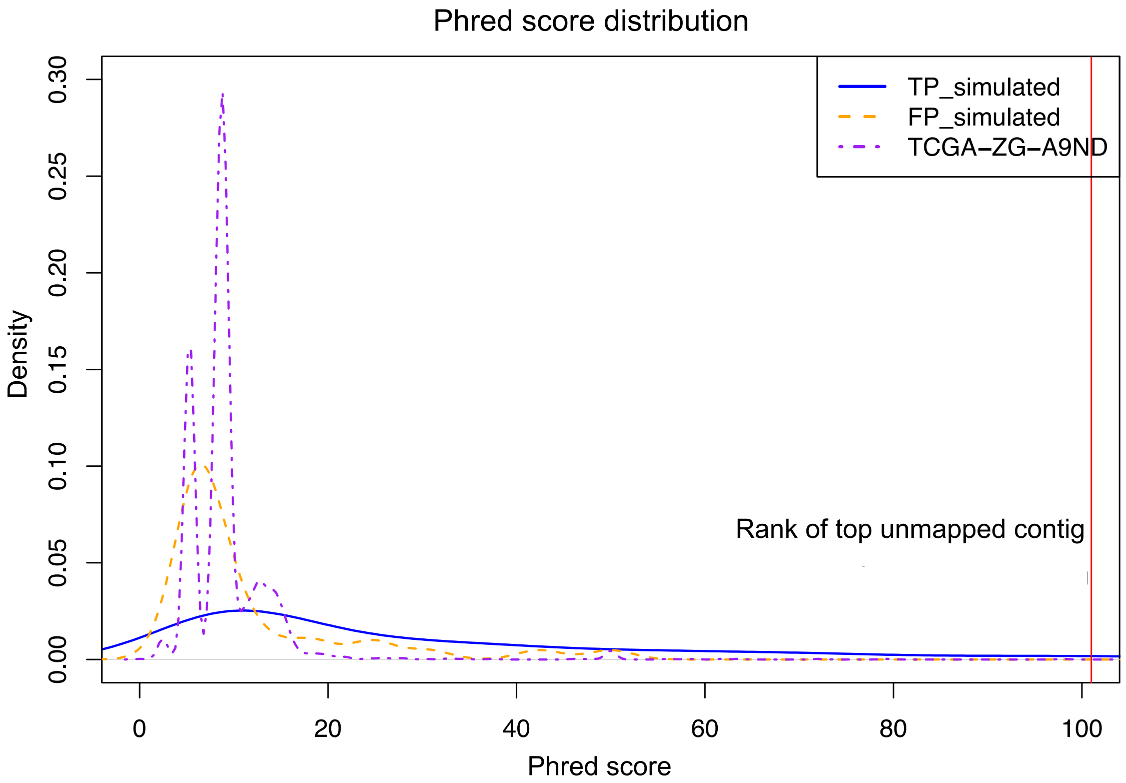

Figure S2. 2-kupl Phred score distribution. *The x axis represents Phred scores calculated by 2-kupl for a simulated WES dataset (TP and FP calls) and for TCGA-PRAD patient TCGA-ZG-A9ND. The red vertical line represents the rank of the top unmapped event detected by 2-kupl in the same patient.*

```
GGGAGGGGTACTGTCACTCTGTTGCAGTAACAAGTTGCAAAATCTTCAGGCTGCAGGCTGCT
|||||||||||||||||||||||||||||.||||||||||||||||||||||||||||||||
GGGAGGGGTACTGTCACTCTGTTGCAGTAATAAGTTGCAAAATCTTCAGGCTGCAGGCTGCT
Score=302.5    47       112       0         88
```

Figure S3 Alignment of a mutant contig and its inferred reference as shown in 2-kupl output. *The event is from TCGA-PRAD patient TCGA-ZG-A9ND. Alignment score is calculated by Biopython. The following statistics are estimated supporting reads and coverage in case and control, respectively.*

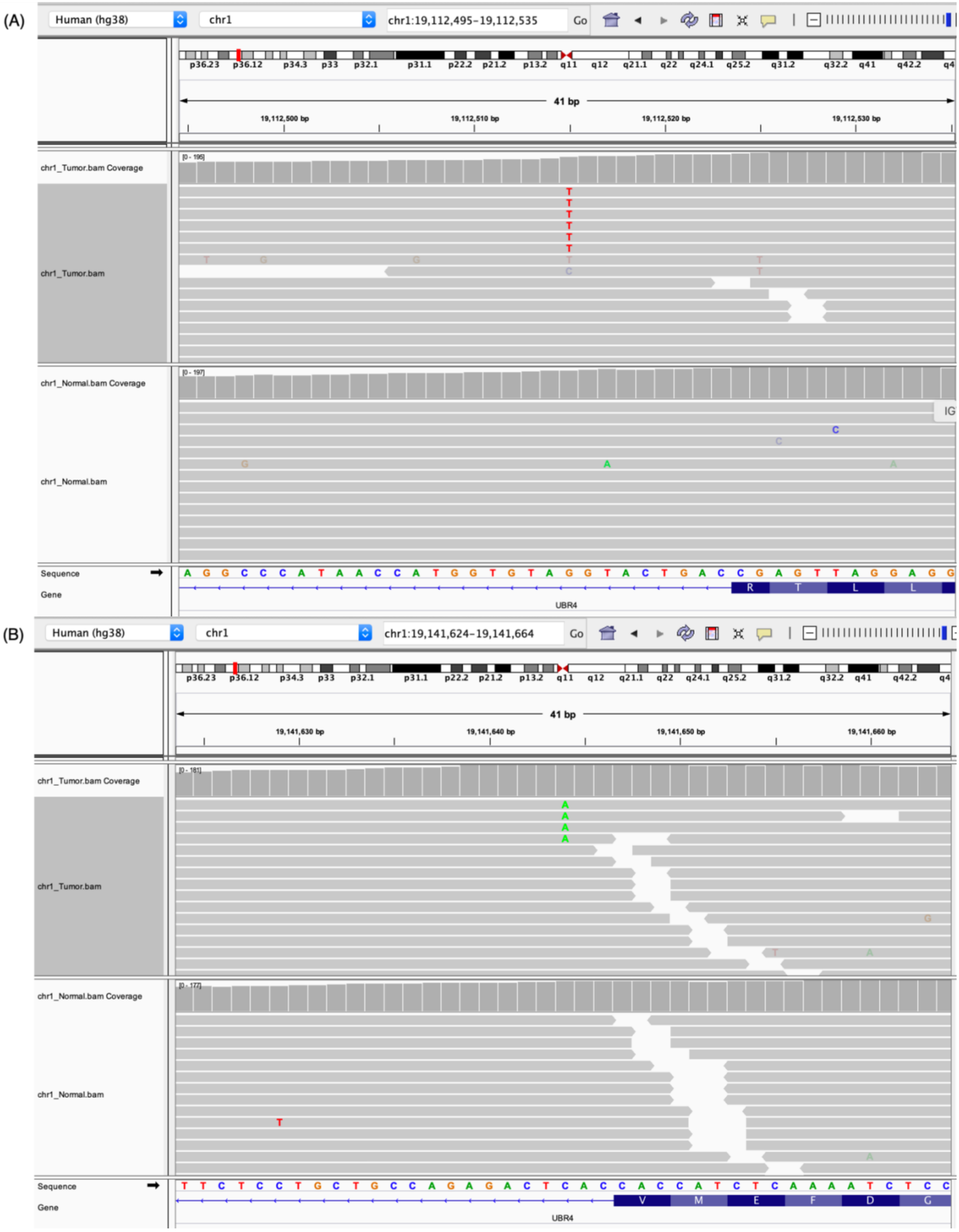
(A)
Human (hg38)
chr1
chr1:19,112,495-19,112,535
41 bp
19,112,500 bp
19,112,510 bp
19,112,520 bp
19,112,530 bp
chr1_Tumor.bam Coverage
chr1_Tumor.bam
chr1_Normal.bam Coverage
chr1_Normal.bam
Sequence
Gene
UBR4
(B)
Human (hg38)
chr1
chr1:19,141,624-19,141,664
41 bp
19,141,630 bp
19,141,640 bp
19,141,650 bp
19,141,660 bp
chr1_Tumor.bam Coverage
chr1_Tumor.bam
chr1_Normal.bam Coverage
chr1_Normal.bam
Sequence
Gene
UBR4

(C)

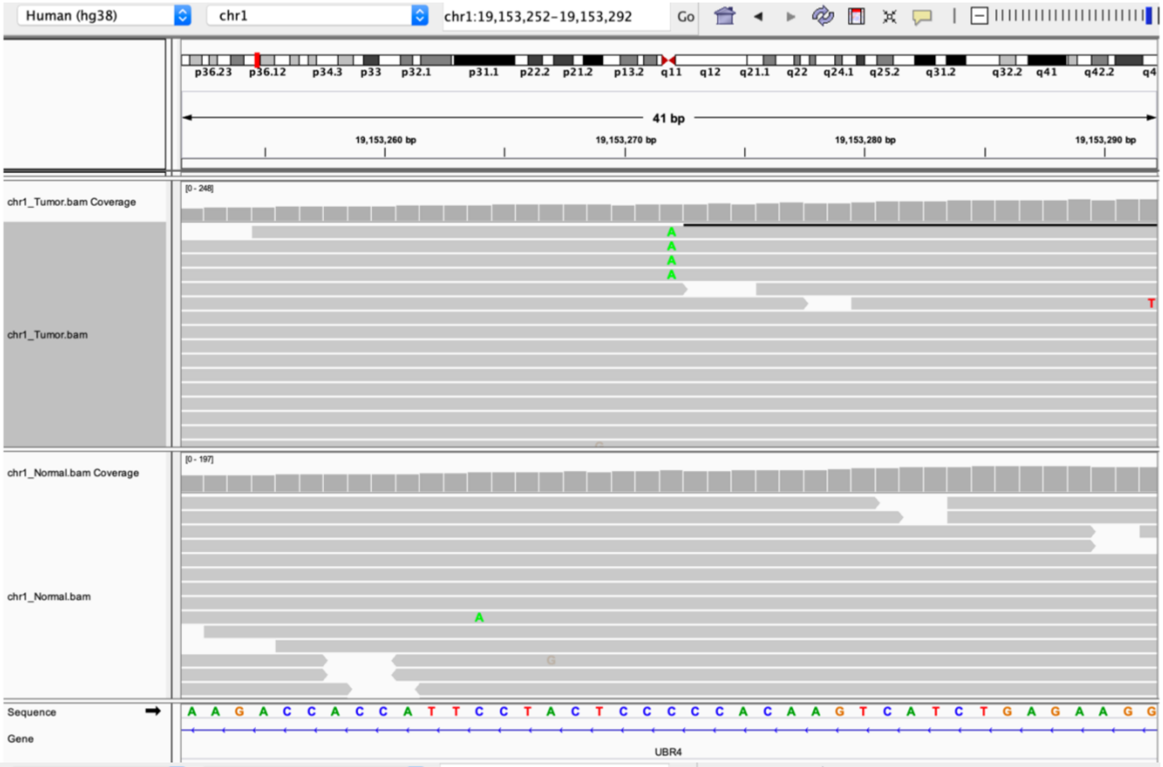
Human (hg38)
chr1
chr1:19,153,252-19,153,292
Go
p36.23 p36.12 p34.3 p33 p32.1 p31.1 p22.2 p21.2 p13.2 q11 q12 q21.1 q22 q24.1 q25.2 q31.2 q32.2 q41 q42.2 q4
41 bp
19,153,260 bp
19,153,270 bp
19,153,280 bp
19,153,290 bp
chr1_Tumor.bam Coverage
[0 - 248]
chr1_Tumor.bam
chr1_Normal.bam Coverage
[0 - 197]
chr1_Normal.bam
Sequence
A A G A C C A C C A T T C C T A C T C C C C C A C A A G T C A T C T G A G A A G G
Gene
UBR4

(D)

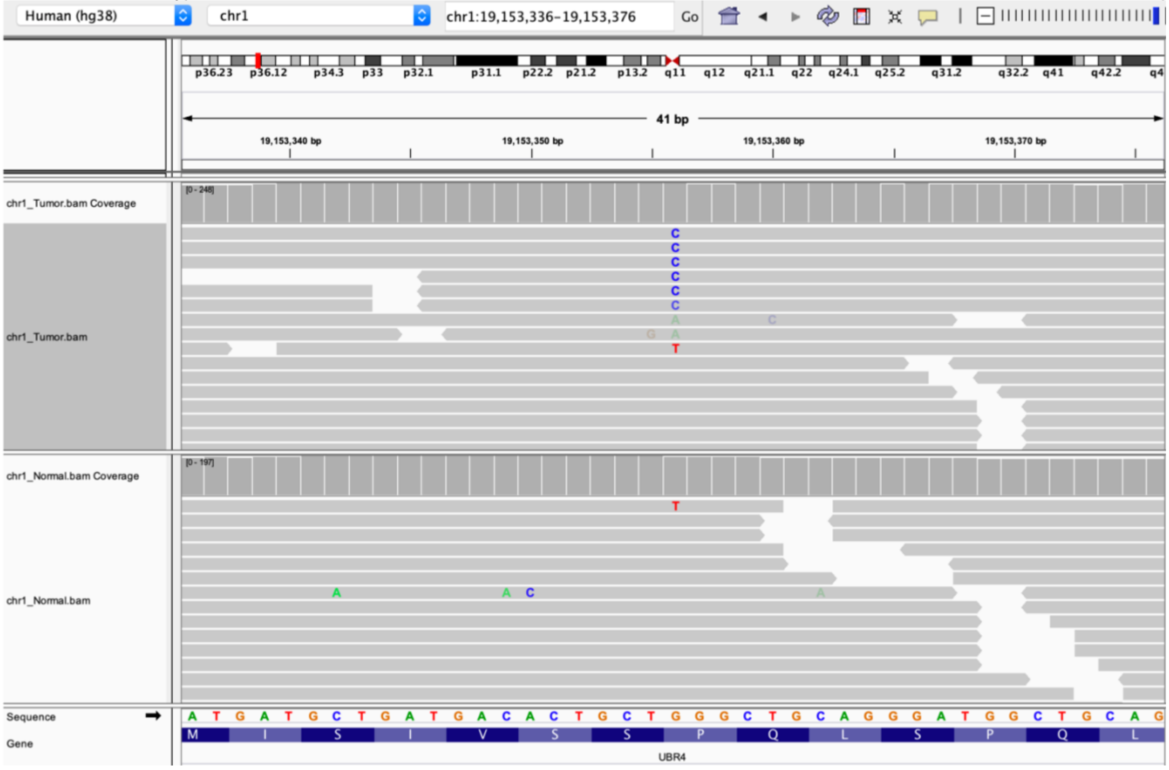
Human (hg38)
chr1
chr1:19,153,336-19,153,376
Go
p36.23 p36.12 p34.3 p33 p32.1 p31.1 p22.2 p21.2 p13.2 q11 q12 q21.1 q22 q24.1 q25.2 q31.2 q32.2 q41 q42.2 q4
41 bp
19,153,340 bp
19,153,350 bp
19,153,360 bp
19,153,370 bp
chr1_Tumor.bam Coverage
[0 - 248]
chr1_Tumor.bam
chr1_Normal.bam Coverage
[0 - 197]
chr1_Normal.bam
Sequence
A T G A T G C T G A T G A C A C T G C T G G G C T G C A G G G A T G G C T G C A G
Gene
M I S I V S S P Q L S P Q L
UBR4

(E)

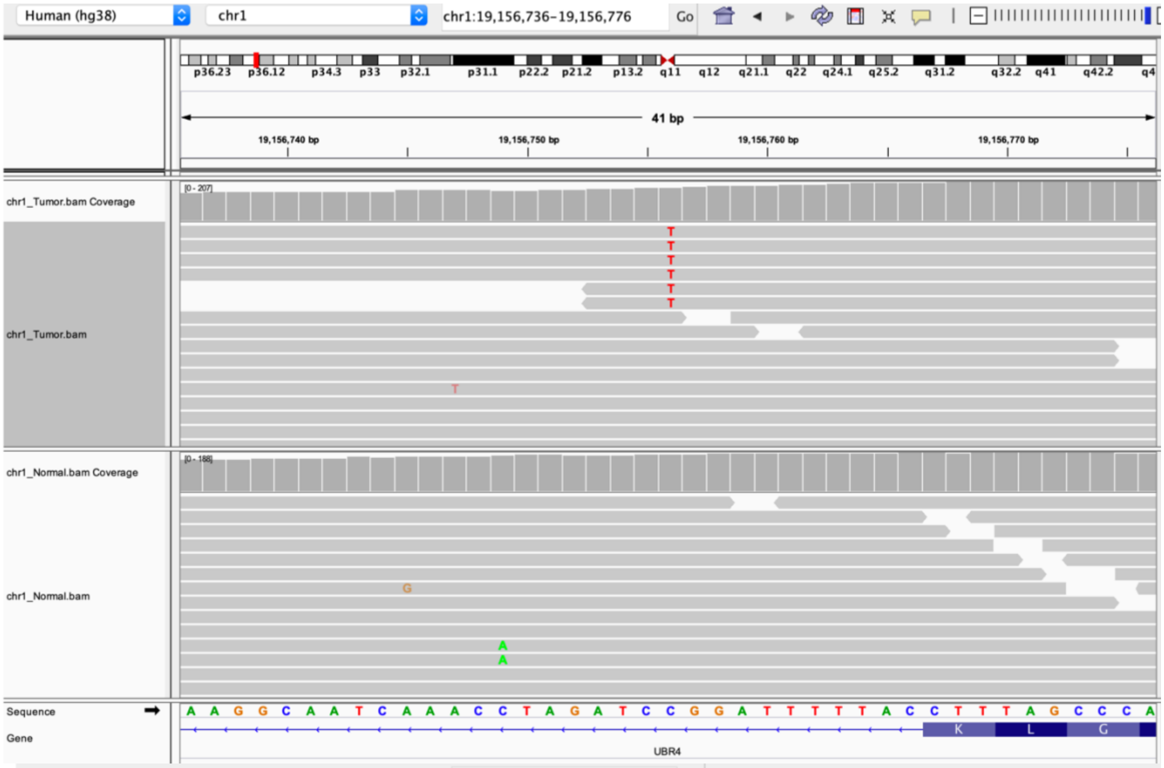
Human (hg38)
chr1
chr1:19,156,736-19,156,776
Go
41 bp
19,156,740 bp
19,156,750 bp
19,156,760 bp
19,156,770 bp
chr1_Tumor.bam Coverage
chr1_Tumor.bam
chr1_Normal.bam Coverage
chr1_Normal.bam
Sequence
Gene
UBR4

(F)

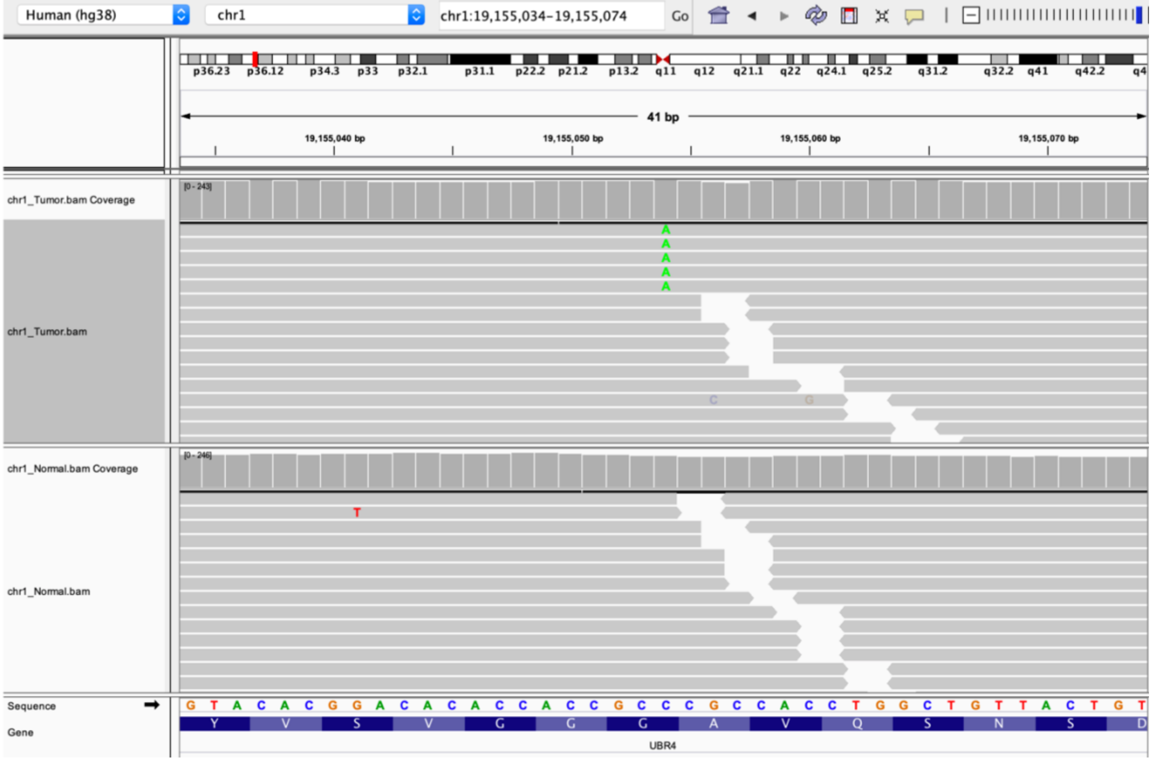
Human (hg38)
chr1
chr1:19,155,034-19,155,074
Go
41 bp
19,155,040 bp
19,155,050 bp
19,155,060 bp
19,155,070 bp
chr1_Tumor.bam Coverage
chr1_Tumor.bam
chr1_Normal.bam Coverage
chr1_Normal.bam
Sequence
Gene
UBR4

(G)

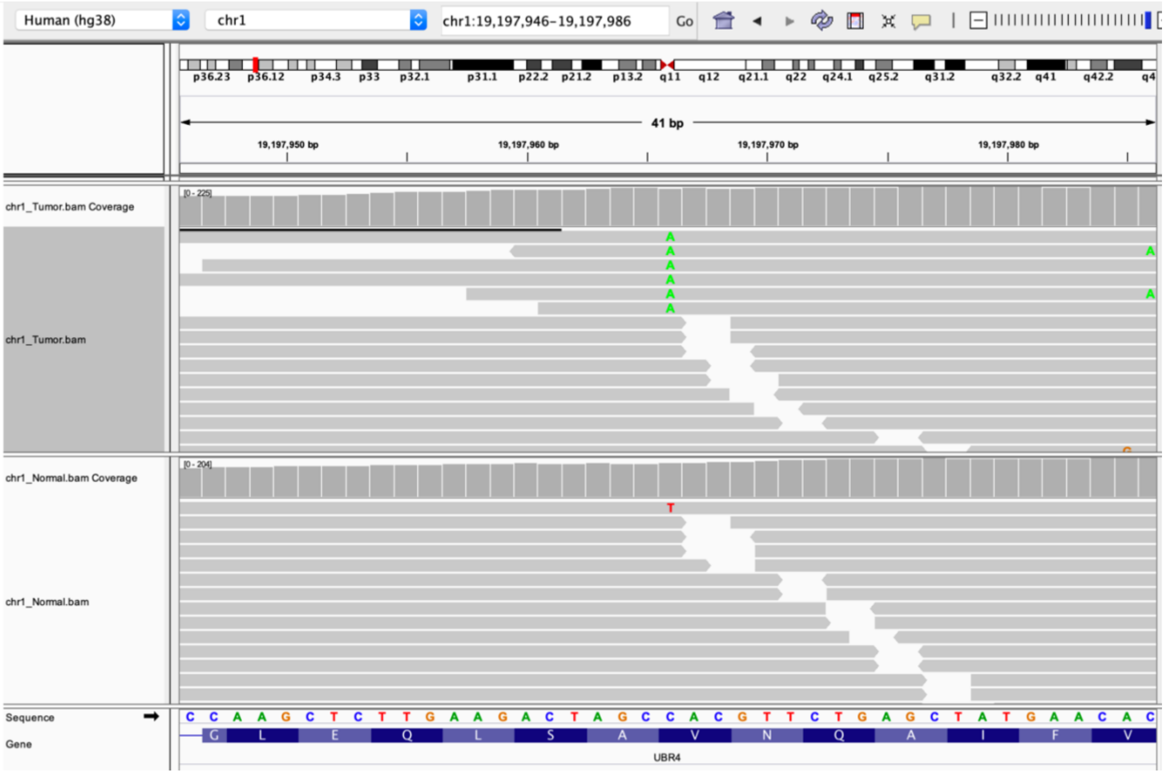

Figure S4. IGV views of UBR4 mutations found in patient of TCGA-EJ-7125. *Panels A-G show seven UBR4 somatic mutations that are absent in GDC portal variants. Top and bottom tracks show aligned reads from the tumor and matched normal library, respectively.*
